# Cryoablation for the Treatment of Kidney Cancer: Comparison With Other Treatment Modalities and Review of Current Treatment

**DOI:** 10.7759/cureus.31195

**Published:** 2022-11-07

**Authors:** Charles A Bisbee, Jeremy Zhang, Justin Owens, Syed Hussain

**Affiliations:** 1 Radiology, HCA Florida Osceola Hospital, Kissimmee, USA; 2 Interventional Radiology, HCA Florida Osceola Hospital, Kissimmee, USA

**Keywords:** radio-frequency ablation, radical nephrectomy, partial nephrectomy, percutaneous cryoablation, renal cancer, renal cell carcinoma (rcc)

## Abstract

With cancer among the leading causes of death worldwide and kidney cancer among the more common cancers in the United States, it has become increasingly important to ensure that first-line treatments remain validated and supported in recent literature. Surgical intervention has long remained the gold standard for intervention but with newer techniques and technology on the horizon, there must be a constant review of other options that may provide improved outcomes and reduction of associated risks. Ablative techniques have gained traction and are becoming a valuable intervention for multiple different types of cancers, kidney cancer included. Cryoablation, a newer ablative technique taking advantage of extreme cold to freeze and destroy abnormal tissue, provides a promising option for treatment. Currently, no review article, to our knowledge, compares all the different treatment options for kidney cancer. Additionally, while some literature has addressed cryoablation in comparison to other methods of management, there has not been an extensive review to combine our current understanding of these comparisons. In this review article, we provide an overview of each of the commonly used treatments for kidney cancer and summarize the current literature regarding the advantages and disadvantages of each intervention. Finally, we seek to compare cryoablation, a newer option for treatment, to each of the approaches with the goal of evaluating the best methods for management and determining cryoablation's role alongside these current interventions.

## Introduction and background

Kidney cancer has nearly doubled in incidence over the past half-decade and currently exists as one of the top 10 most common neoplasms in the US [1]. Of the different tumors of the kidney and renal pelvis, renal cell carcinoma (RCC) accounts for the vast majority, comprising over 90% of kidney tumors [2]. Like many other neoplasms, risk factors for kidney cancer include exposure to tobacco smoking and obesity. Chronic renal failure, elevated blood pressure, and exposure to chemicals such as trichloroethylene can also contribute [3]. While there may be slight differences in the treatment approach depending on the type or stage of a renal neoplasm, the general approach to cancer of the kidney remains the same. Current therapy ranges from medical management with cytokine therapy or immunotherapy, to surgical intervention such as partial nephrectomy or radical nephrectomy in which a region of the kidney or the whole kidney is removed.

Over the past few decades, given advances in modern imaging, minimally invasive ablative procedures, such as cryoablation, have gained traction and are increasingly being used in treatment [4]. Cryoablation in kidney cancer is a percutaneous method during which cryoprobes are inserted into the malignant tissue under guidance from computed tomography (CT) imaging. After proper positioning is confirmed, argon gas is circulated through the cryoprobe resulting in rapid cooling to temperatures and freezing of the surrounding tissue through conduction. Temperatures below -20 degrees Celsius are sufficient to cause cell death of normal renal parenchyma; however, some cryo-resistant tumor tissue may require temperatures at or below -40 degrees Celsius to ensure complete destruction [5]. The rapid cooling/thawing cycle destroys cancerous tissue around the probe through direct mechanisms with cold-induced physical damage and indirect mechanisms involving metabolic disruptions within the cell [6]. Direct mechanisms include cold-induced vessel thrombosis leading to ischemic death along with the initiation of cellular apoptosis and low temperatures [7]. The indirect mechanism involves a cycle that is unique to cryoablation which results in intra- and extra-cellular ice crystal formation that results in cellular membrane disruption and cell death [7].

Cryoablation has proven itself to be an effective approach for those seeking to avoid more invasive surgical methods, with several studies demonstrating its high efficacy [8-10]. Focal therapy with cryoablation can preserve renal function, decrease bleeding risk/time, and decrease the length of stay, all desirable outcomes contributing to the procedure’s rising popularity. The procedure is also associated with shorter operating times [11]. Despite evidence supporting cryotherapy, current guidelines, notably those from the American Urological Association (AUA) and the European Association of Urology (EAU), recommend partial nephrectomy for renal masses <4 cm and advanced metastatic disease often requires medical management or radiation therapy (RT) to slow progression [12,13]. Given its relatively new application, it is important to discuss the advantages and disadvantages that cryoablation has when compared to more traditional treatment modalities. The purpose of this review is to summarize the current literature surrounding widely used kidney cancer treatments while also comparing these treatments with a newer treatment, cryoablation, to reassess the best methods for management.

## Review

Partial/radical nephrectomy

History and Current Trends

The use of nephrectomy for the purpose of removing a mass in the kidney has had a long history with the radical nephrectomy first described in 1963, and the first partial nephrectomy used to remove a kidney tumor dating back to 1887 [14,15]. Since then, nephrectomies, both radical and partial, have become hallmark procedures used for the treatment of kidney cancer. Radical nephrectomy involves surgical removal of the entire kidney, including the surrounding fat. Occasionally, the adrenal gland and the paraaortic and para-caval lymph nodes are also removed although there are limited data to support routine lymph node dissection, and adrenalectomy is not associated with better oncological outcomes [16,17]. A partial nephrectomy takes a more localized approach, during which only the diseased portion of the kidney is removed, with the benefit of preserving kidney function. Historically, radical nephrectomy was the chosen standard of care when treating renal masses. Advances in surgical treatments and research have proved that partial nephrectomy provides similar oncological outcomes compared to radical nephrectomy in small renal masses [18]. There has been an increased push for nephron-sparing approaches in an attempt to preserve kidney function.

Indications

Indications for partial nephrectomy have been expanded over the years and increased from individuals with imperative indications such as bilateral tumors or advanced renal insufficiency to all individuals with kidney masses less than 4 cm in diameter [19]. Current guidelines recommend partial nephrectomy as the treatment of choice for kidney masses <4 cm in diameter and limited to the kidney (RCC stage cT1a) in an effort to prioritize nephron-sparing approaches, and can be done independently of tumor size whenever technically feasible [19,20]. Radical nephrectomy is indicated only in the presence of certain conditions in the setting of a highly complex kidney tumor. These conditions include tumors with malignant and rapid growth potential, the lack of pre-existing chronic kidney disease (CKD), and the presence of a normal contralateral kidney that will sustain a baseline glomerular filtration rate (GFR) >45 ml/min post-nephrectomy [19].

Advantages

Both of these procedures can be done open, laparoscopically, or robot-assisted. Laparoscopic and robot-assisted techniques are associated with better perioperative advantages, including shorter length of stay, fewer overall complications, and lower estimated blood loss [21]. Studies have shown that radical nephrectomy may decrease overall survival, as its association with cardiovascular morbidity and mortality can be linked to increased risks of renal failure compared to partial nephrectomy [22]. Partial nephrectomy has a large advantage over radical nephrectomy based on the nephron-sparing approach thus reducing the risk of developing CKD. One study found that radical nephrectomy has up to a 65% chance of CKD development. In comparison, the risk with partial nephrectomy is as high as 20% [23].

Disadvantages

These surgical interventions are not without limitations, rendering patients anephric or at high risk of renal replacement therapy due to CKD. Partial nephrectomy is associated with an average of 20% decline in kidney function relative to the pre-surgical baseline [24]. Patients undergoing radical nephrectomy are much more likely to develop CKD, up to 65% [23]. Current surgical approaches and indications are optimized to prioritize preserving renal function by optimizing nephron preservation.

Radiofrequency ablation

History and Current Trends

The idea of radiofrequency ablation (RFA) has been around since 1931, and RF energy has been used for catheter ablation of various tissue types for over 30 years now [25]. Initially gaining popularity in the use of cardiac ablation to treat arrhythmias, RFA has gained traction in the removal of many unwanted tissues throughout the human anatomy. However, it is still a relatively new technique in the case of kidney tumor ablation that has not gained a reputation and popularity similar to that of the current gold standard, partial nephrectomy [26]. With recent trends toward less invasive procedures when appropriate, RFA is a potentially promising alternative to the more invasive partial and radical nephrectomy options that have been the standard of care for many years.

Technique 

RFA of kidney tumors is done using image-guided probes that are inserted into the tumor and use high-frequency electrical current that leads to tissue destruction through heat energy. These probes use monopolar alternating currents of 400-500 kHz to increase the surrounding temperature of the probe to above 70 degrees Celsius, necessary to cause coagulation necrosis of the desired tissue [26]. Some manufacturers recommend maintaining a temperature greater than 100 degrees Celsius for 8 mins to ensure tissue necrosis. 

Effective tumor ablation is achieved through the optimization of heat production and duration to minimize heat loss. While heat production is correlated with the intensity and duration of the RF energy deposited, heat loss is mainly due to the blood flow within adjacent blood vessels [27]. Rapid loss of heat occurs at a determined distance from the probe, which limits the total size of ablation possible through RF energy. This is an important aspect to consider with tumor ablation because of the need for safe ablation margins. Currently, recommendations are approximately 1 cm safe ablation margins for most tumors [27].

Indications

RFA is a useful tool and is applicable in multiple disciplines throughout medicine. It has been used for, but is not limited to, peripheral nerve pain, cardiac arrhythmias, chronic pain from various arthritic diseases, and tumor masses [27]. The use of RFA with respect to kidney tumors is generally reserved for patients that can not tolerate general anesthesia, high risk for surgical complications due to comorbidities/age, recurrent tumor after surgical resection, or small tumors with a functional solitary kidney [26,27]. Currently, the AUA recommends RFA or cryoablation as an alternative approach for the management of cT1a solid kidney masses <3 cm in size with evidence Grade C [19].

Advantages

When comparing RFA with partial and radical nephrectomy, RFA provides distinct advantages in total complication rates without significantly changing oncological outcomes [28,29]. In a previous literature review done by Wen et.al, there was a failure rate of 7% in tumor ablation and a complication rate of 16% found from a total of 425 tumor removals with a follow-up of one year [29]. When compared to partial nephrectomy in a three-year actuarial analysis, it’s shown that RFA for cT1a kidney tumors has similar oncological outcomes [30]. Recent studies have shown renal ablation compared to partial nephrectomy leads to preserved renal function, decreased bleeding risk/time, decreased length of operation, and decreased length of stay [11]. Another study comparing RFA and partial nephrectomy determined that oncological and functional outcomes were similar and that both are safe and effective procedures for small tumors in a solitary kidney [28]. RFA continues to be a great alternative for patients that are at high risk, given their age or comorbidities, for more invasive procedures that require general anesthesia and can lead to more postoperative complications.

Disadvantages

A pitfall to RFA is the potential for tissue charring at temperatures above 100 degrees Celsius. This charred tissue creates a layer of insulation around the probe that prevents the peripheral tissue from receiving the ablative RF energy required for further coagulative necrosis [26,27]. Without the appropriate distribution of energy to create effective ablation margins, there will be a higher risk of residual cancerous tissue, which leads to a high chance of recurrence. Another obstacle with the RFA procedure involves the pre- and post-ablation imaging done to confirm acceptable margins and survey for any residual tissue. CT imaging is most commonly used to review post-procedure results. Due to post-ablation changes to the surrounding tissue and patient respirations/motion, there is variability in the anatomy between each scan, which makes it difficult to accurately estimate the safety margins [31]. These two main disadvantages of RFA are avoided in percutaneous cryoablation and the other treatment modalities for renal tumors discussed in this review.

Radiation therapy

History and Current Trends

Radiation therapy (RT) in RCC has mainly been reserved for treatment when metastasis was confirmed under the assumption that kidney tumors are radioresistant. Even in the setting of neoadjuvant RT for RCC, studies have failed to show the benefit of RT prior to nephrectomy when compared to nephrectomy alone. The study showed no significant difference in five-year survival when looking at a series of 126 non-metastasized RCCs [32,33]. However, with the advancement of RT technology like stereotactic radiation, in which hypofractionated RT is delivered over a few sessions to attain tumor ablation, RT is now another potential treatment modality for non-surgical patients that have small kidney tumors like RCC [34].

Indications

Patients with larger RCC tumors, such as those classified as T1b or greater, are often limited in their treatment options given a decreased effectiveness with thermal ablation in this setting. This is often a result of an increased risk of local recurrence and complications [35,36]. In patients who are poor surgical candidates, such as elderly patients, those on long-term anticoagulation, or those with multiple competing comorbidities, stereotactic body radiation therapy (SBRT) provides a viable alternative [35,36]. A systematic review and meta-analysis found that kidney radiation therapy is locally effective and associated with low toxicity rates for primary RCC with the patients in these studies deemed inoperable with more invasive procedures [34]. Given its noninvasive nature, SBRT has been increasingly used in metastatic RCC and may be helpful in both metastatic disease to the brain and spine as well as extra-cranial metastatic disease [36]. SBRT may also be indicated alongside systemic treatment to provide a potential additive effect [36]. Currently, AUA reports that the use of SBRT for kidney tumors is still investigational and limited to patients that remain inoperable for surgery or other minimally invasive procedures, which is supported by the current European Association of Urology and European Society of Medical Oncology guidelines [19,36]. The 2022 National Comprehensive Cancer Network version 1.0 kidney cancer guidelines add additional recommendations for SBRT, expanding recommendations for SBRT as a treatment option for metastatic sites in selected patients with oligometastatic RCC, or in cases treated with immunotherapy or targeted therapy [36].

Advantages

RT provides an advantage when surgery is not indicated. One article conducted a population-based cohort study and found that SBRT led to longer survival compared to observation alone [37]. In patients with primary RCC, SBRT was observed to have a local control rate of above 90% with only a 0-9% chance of grades 3-4 toxicity [36]. SBRT has also been shown to provide relief of symptoms associated with metastatic disease from kidney malignancy. Treatment for metastases to bone and brain can provide local symptom control and is the most common use for RT in kidney malignancy management currently [13]. In a study of 1225 patients with intracranial metastatic RCC, this local control rate ranged between 90 and 97% with SBRT [36]. SBRT has also shown benefits in oligometastatic disease and was observed to delay the need for systemic therapy for at least 1 year in 70-90% of patients while still maintaining low toxicity [36]. Another advantage of SBRT is that it is non-invasive, so complications from surgical procedures like bleeding, postoperative infections, general anesthesia, etc. are avoided. While systemic treatment is currently the frontline treatment for metastatic disease, SBRT also has been observed to be both safe and feasible when used alongside systemic therapy. The combination of these two treatments may exploit a synergistic interaction between the modalities to increase response to therapy [36].

Disadvantages

One aspect of concern for SBRT is its dose-limiting toxicity. It is difficult to achieve a high enough radiation dose to sufficiently treat the cancerous tissue before inducing symptoms that are not tolerable to the patient like intractable abdominal pain, nausea, and vomiting. One study has shown dose escalation to 48 Gy was successfully achieved without reaching dose-limiting toxicities [38]. Although this is only a phase I trial, further studies are needed to confirm the optimal dosing for renal tumors. At present, there is not a large enough body of evidence and further research is needed given the novelty of this application for early-stage kidney tumors. Additionally, a poorer prognosis is seen in patients with larger tumor sizes, higher baseline creatinine, and worse performance status despite the use of SBRT [35]. Given the slow-growing nature of RCC, the tumor can also be expected to show a slow radiographic response [36].

Medical management

History and Current Trends

Medical management in kidney cancer has evolved over the past few decades as advancements in targeted therapy and immunotherapy have provided more effective drugs for treatment. Prior to these advancements, starting in the late 1980s, cytokine therapy was considered the cornerstone of medical management. Agents such as interferon alfa and high-dose interleukin-2 were among the most evaluated cytokine treatments, although studies found these drugs were associated with a low overall response rate and provided marginal survival advantages. Additionally, these drugs were often associated with severe toxic side effects that limited use [39]. Currently, medicinal treatment focuses on the use of newer anti-angiogenic agents such as vascular endothelial growth factor (VEGF) tyrosine kinase inhibitors (TKIs), mammalian target of rapamycin (mTOR) inhibitors, and immunotherapy [40]. While an effective approach for other forms of cancer, chemotherapy has a limited role in the treatment of kidney cancer, often only used in situations in which targeted therapy and immunotherapy options have been exhausted [41]. Despite some trials indicating responding patients survive longer with chemotherapy, no clear survival benefit has been found for patients with advanced RCC undergoing chemotherapy treatment [41].

Anti-angiogenic agents such as vascular endothelial growth factor tyrosine kinase inhibitors (VEGF-TKIs) work through the blockade of a cell signaling pathway involved in the formation of new blood vessels. Interruption to this pathway cuts off the nutritional pathway of the tumor cells and limits growth in the process. Examples include sorafenib, sunitinib, pazopanib, and axitinib. mTOR inhibitors, such as rapamycin and everolimus, similarly work to kill cancer cells as mTOR plays a pivotal role in governing cell growth and proliferation [42]. With developments in immunotherapy, however, these drugs may soon begin to be considered the second line. Immunotherapy with T-cell checkpoint inhibitors (TCCIs) that target programmed cell death 1 (PD-1) and cytotoxic T-lymphocyte-associated antigen 4 (CTLA4) receptors, leads to an increased anticancer response by effector T-cells through blockade of this pathway [13].

Indications

While invasive options, such as partial nephrectomy, are indicated and potentially curative in the treatment of smaller, localized masses, it is not often a viable option in patients presenting with advanced metastatic disease such as stage IV RCC. This can be the case for approximately 30% of patients who present with metastatic RCC at the time of diagnosis or 40% of patients who experience recurrence of their disease following initial management with surgery [40]. In these situations, medical management is often the choice of treatment to further slow the progression of the disease.

The International Metastatic Renal Cell Cancer Database Consortium (IMDC) provides additional indications for drugs and combinations of systemic therapy in Stage IV RCC based on whether the patient falls into a favorable risk or intermediate/poor risk category [43]. For patients with earlier stages of kidney cancer, primarily Stages II and III of RCC, while medical management is not effective alone, drugs such as pembrolizumab, a PD-1 inhibitor, can sometimes be indicated in combination with radical nephrectomy for treatment [44].

Advantages

Medical management may provide an advantage over surgical treatments in advanced metastatic disease where removal of the tumor alone is insufficient. While cytokine therapy has largely been replaced by more novel treatments, one advantage of high-dose interleukin-2 over other options is its status as the only curative systemic therapy for RCC [45]. Despite this advantage, among drugs used for systemic therapy, drugs such as VEGF TKIs and mTOR inhibitors have ultimately been found to have a net advantage over cytokine treatment due to a higher overall response rate and a more favorable safety profile [13].

Nivolumab, a PD-1 inhibitor, has been shown to improve overall survival, and when compared to everolimus a clear advantage for overall survival was seen for nivolumab [41]. Additionally, Motzer studied the dose-response relationship for three different doses of nivolumab (0.3, 2, and 10 mg/kg) and found that nivolumab had a manageable safety profile while still demonstrating antitumor activity. Of the treatment-related adverse events that did occur with nivolumab, they were generally low-grade in severity [46]. Finally, for Stages II and III RCC, Choueiri et al. observed that pembrolizumab had advantages as an adjuvant treatment alongside radical nephrectomy and was associated with a higher disease-free survival (77.3%) compared to a placebo (68.1%) [44].

Disadvantages

The use of systemic therapy for the treatment of advanced metastatic disease comes with some notable disadvantages. Primarily, many of the systemic drugs used in the treatment of metastatic renal cell carcinoma have been found to have associated toxicities. Cytokine therapy with high-dose interleukin-2 can only be selectively used, as it requires patients to have an excellent performance status and no cardiorespiratory comorbidities because of its high toxicity profile. VEGF-TKI treatments, while less toxic than cytokine therapy, are associated with numerous side effects. Hypertension is a well-recognized side effect seen in 17-40% of users, and up to 70% of patients undergoing treatment with TKIs had some sort of thyroid abnormality, with other side effects such as diarrhea reported as well [13]. Immunomodulators such as PD-1 inhibitors have side effects that are autoimmune in origin and can affect any organ with the pulmonary, endocrine, gastrointestinal organs, and skin most commonly impacted [13]. Pembrolizumab, for example, an option alongside radical nephrectomy, had 20% of patients report serious adverse events in one study, the most common being diarrhea, fatigue, and rash [44].

Outside of the toxicities associated with systemic therapy, the disadvantages of the drug include a number of gaps in the management of kidney cancers such as RCC. Greef outlined some of these gaps noting that for early-stage diseases, medical management is not often a viable option, and while pembrolizumab has shown some efficacy alongside radical nephrectomy, there are few occurrences of other effective adjuvant treatments with systemic therapy [13,44]. Also, despite advancements in systemic treatment, not all patients respond to therapy and the median survival of patients with metastatic RCC remains under three years [13].

Discussion

Cryoablation Compared to Invasive Techniques

As a newer method for the treatment of kidney cancer, cryoablation has shown potential. Literature regarding thermal ablation therapies for the management of small kidney masses (<3 cm) has shown promising results. However, an Agency for Healthcare Research and Quality's (AHRQ's) meta-analysis comparing partial nephrectomy and thermal ablation therapies found similar cancer-specific survival across both therapies with no single, superior treatment modality [47]. When comparing overall survival, partial nephrectomy demonstrated superiority over thermal ablation [47]. This may be rooted in selection bias for each of the respective therapies with healthier patients suitable for surgical therapies, as well as patient-specific comorbidities and competing risks for mortality. Individual risk factors remain an essential aspect of determining what treatment strategy will be best for the patient. Current AUA guidelines place thermal ablation as a moderate recommendation with grade C evidence level for appropriate patients [19].

When comparing thermal ablation to radiofrequency ablation, there have been no significant differences in treatment outcomes defined by complications, metastatic progression, or cancer-specific survival [48]. However, in one multicenter study comparing RFA and cryoablation (CA), overall five-year survival rates in a total of 46 patients were 78% and 82%, respectively, after tumor ablation of RCC >4 cm but <7 cm (stage cT1b) [49]. The study found that the primary efficacy rate was significantly higher in cryoablation compared to RFA. Primary efficacy was defined as complete coverage of the index tumor by the ablative zone without signs of residual tumor following the initial procedure [49]. One of the distinct advantages of cryoablation over RFA is that it can be continuously monitored throughout the procedure to ensure sufficient tumor coverage and appropriate margins. Studies have shown that RFA required more ablations to provide sufficient tumor ablation margins vs cryoablation [4,26,49,50]. Another benefit of cryoablation is the ability to have greater shape control of the ablation zone when needed [4,51]. This has much to do with the potential for charring given the inherent biophysics of RF energy ablation. There is also a study that analyzed a total of 10 studies with over 2,000 patients that showed an association with lower local recurrence rates for cryoablation when compared to RFA [52].

Multiple studies have shown comparable oncologic efficacy between thermal ablation and nephrectomy of cT1a kidney tumors while preserving renal function [28,53-55]. On the other hand, when looking at tumors 2-4 cm in size, another study analyzed over 17,234 patients that underwent treatment for RCCs <4 cm in size suggesting RFA and cryoablation is not as effective as partial nephrectomy when comparing overall survival in masses 2-4 cm in size, with RFA vs partial nephrectomy hazard ratio (HR) of 2.99 and cryoablation vs partial nephrectomy HR of 2.14 [56]. For RCCs sized 2 cm or less, RFA still showed lower overall survival, HR 2, but cryoablation showed no significant difference in overall survival compared to PN, HR 1.3 with a confidence interval of 0.90-1.96 [56]. Even with similar oncological outcomes, it seems that RFA is not as effective for overall prognosis as cryoablation when compared to the current standard of treatment, partial nephrectomy.

Cryoablation Compared to Non-invasive Options

There are very few articles that have done a direct comparison of SBRT vs cryoablation and should be researched further once the evaluation of the efficacy of SBRT is complete. Radiation therapy is often used in situations in which patients are unfit for surgery compared to cryoablation, which has been found to have a strong benefit in focal cancers due to a low recurrence and failure rate [5,57]. The role of radiotherapy in primary kidney cancer is questionable and while SBRT may show promise, additional research is necessary to clarify its role. Comparison is limited by a lack of literature, however, there may be benefits in combined therapy with radiation therapy used alongside cryoablation. For example, in hepatic malignancies, cryoablation has been shown to be implemented alongside intensity-modulated radiation therapy (IMRT) safely, although this study was limited by a small sample size [58]. Given its implementation in hepatic cancer, there may be similar value in using radiotherapy alongside surgical or ablative techniques. For localized renal cell carcinomas, however, cryoablation appears to provide a more logical response given its better outcomes in this setting.

As for medical management, comparing cryoablation and immunotherapy is again limited, as these two approaches are generally used in the treatment of two different stages of kidney disease. Cryoablation serves as an option for earlier stages of kidney cancer that is locally contained, whereas immunotherapy and other medical management are relied upon in metastatic disease where surgery or invasive treatment is no longer an option. However, combination therapy may again be supported in this instance by evidence showing the efficacy of cryoablation use in metastatic disease alongside systemic therapy. A review by Aarts showed that 41 of 45 publications demonstrated favorable effects for cryoablation when combined with other therapies leading to a potential enhancement of the anti-cancer immune response [59]. Other studies have shown that multisite cryoablation was associated with low morbidity and local tumor recurrence rates at all anatomic sites tested and an increase in overall survival. Therefore, cryoablation as an adjunct to systemic therapies may be a cost-effective option for the palliation of oligometastatic RCC [60].

Risks Associated With Cryoablation

While cryoablation has shown promise, it is important to consider the risks associated with the procedure. Renal hemorrhage is the most significant of complications, although other risks, such as injury to the ureter, infection/abscess, nerve injury, pneumothorax, needle tract seeding, and skin burn, are known to occur [61]. Notably, compared to partial nephrectomy, the current gold standard, the rate of overall and postoperative complications with cryotherapy is lower [62]. Despite the potential complications with cryoablation, the procedure remains a generally safe option, and the majority of complications that occur can often be treated in a conservative manner [61]. Regardless, interventionalists and urologists using percutaneous cryoablation should be aware of the potential risks associated with the procedure and understand techniques to minimize and treat these risks.

## Conclusions

Cryoablation is still a relatively new technique for the treatment of kidney tumors, but with recent studies showing comparable results to current partial nephrectomy treatment options and better efficacy than radiofrequency ablation, it seems that cryoablation should be considered over radiofrequency ablation. Partial nephrectomy remains the gold standard for RCC, and additional comparisons will need to be conducted to determine if thermal ablation can ever replace a surgical approach. In patients in which surgery is contraindicated or those seeking a less invasive approach, cryoablation remains a viable option, however. Comparison with radiation therapy and medical management is limited due to a lack of literature and different indications for these therapies. At this time, the decision to perform a partial nephrectomy or a less invasive alternative should continue to be considered case by case. Ultimately, there is still a need for further research to attain more concrete recommendations for cryoablation.
